# A Review on Splenic Diffuse Red Pulp Small B-Cell Lymphoma

**DOI:** 10.3390/curroncol28060431

**Published:** 2021-12-06

**Authors:** Elif Yilmaz, Arashpreet Chhina, Victor E. Nava, Anita Aggarwal

**Affiliations:** 1 Department of Hematology and Oncology, Georgetown University Medical Center, Washington, DC 20007, USA; elif.yilmaz@medstar.net; 2 Department of Hematology and Oncology, Veterans Affair Medical Center, Washington, DC 20422, USA; arashpreet.chhina@va.gov (A.C.); victor.nava@va.gov (V.E.N.)

**Keywords:** primary splenic lymphoma, splenic diffuse red pulp lymphoma, villous lymphocytes, B-cell lymphoma, non-Hodgkin lymphoma

## Abstract

Splenic diffuse red pulp small B-cell lymphoma (SDRPL) is a rare disease, representing <1% of all non-Hodgkin lymphomas (NHL). The most common clinical manifestations include splenomegaly, lymphocytosis, and hemocytopenia. A diagnosis of SDRPL can be challenging, as it shares multiple clinical and laboratory features with splenic marginal zone lymphoma (SMZL), hairy cell leukemia (HCL), and HCL variant (HCL-v). Obtaining splenic tissue remains the gold standard for diagnosis. In the cases where splenic tissue is not available, diagnosis can be established by a review of peripheral blood and bone marrow studies. SDRPL is characterized by a diffuse involvement of the splenic red pulp by monomorphous small-to-medium sized mature B lymphocytes effacing the white pulp. The characteristic immunophenotype is positive for CD20, DBA.44 (20 to 90%), and IgG, and typically negative for CD5, CD10, CD23, cyclin D1, CD43, annexin A1, CD11c, CD25, CD123, and CD138. The Ki-67 proliferative index is characteristically low. Cyclin D3 is expressed in the majority of SDRPL in contrast with other types of small B-cell lymphomas, thus facilitating the recognition of this disease. There is no standard treatment regimen for SDRPL. Initial treatment options include splenectomy, rituximab monotherapy, or a combination of both. Chemoimmunotherapy should be considered in patients with advanced disease at baseline or progression.

## 1. Introduction

Lymphoid malignancies involving the spleen can be classified as primary or secondary splenic lymphomas, based on the extent and timing of its involvement. Primary splenic lymphomas consist of lymphoid neoplasms that are confined to the spleen and the splenic hilar nodes, without peripheral lymphadenopathy. This group often presents with bone marrow (BM) and peripheral blood (PB) involvement, and occasionally with liver lesions. Secondary splenic lymphomas represent lymphomatous involvement by neoplastic lymphocytes, originating from nodal or extra-nodal stations beyond the splenic hilum. Primary splenic lymphomas are uncommon, representing <2% of all non-Hodgkin lymphomas (NHL). The most common histological types of B-cell primary splenic lymphomas include splenic marginal zone lymphoma (SMZL), hairy cell leukemia (HCL), hairy cell leukemia variant (HCL-v), splenic diffuse red pulp small B-cell lymphoma (SDRPL), and primary splenic diffuse large B-cell lymphoma (PS-DBCL).

SDRPL is a rare type of indolent NHL, composed of small B-lymphocytes that involve the red pulp of the spleen, the bone marrow, and peripheral blood. SDRPL was first recognized as a provisional entity in the 2008 WHO classification of lymphoid neoplasms [1], and is now grouped under splenic B-cell lymphoma/leukemia, unclassifiable per the 2016 revision of the WHO classification [2]. In the older literature, this entity was also defined as SMZL, diffuse variant, SMZL with diffuse red pulp involvement, and splenic red pulp lymphoma with numerous basophilic villous lymphocytes. SDRPL is a diagnosis of exclusion, and the differential includes SMZL, HCL, and HCL-v.

## 2. Epidemiology

The true incidence of SDRPL is unknown. Primary splenic lymphomas in general represent 1–2% of all lymphoid malignancies [3]. SDRPL comprises <10% of B-cell lymphomas that are diagnosed by splenectomy [4,5]. In a single-center case series of 37 patients, SDRPL reportedly represented 0.5% of all chronic lymphoid malignancies that were diagnosed by PB examination [6]. SDRPL has a slight male predominance, with a male/female ratio of 1.5–2.5:1. The majority of the patients are older than 60 years of age, with a median of approximately 65 years [6,7].

## 3. Diagnosis

**Figure 1:**
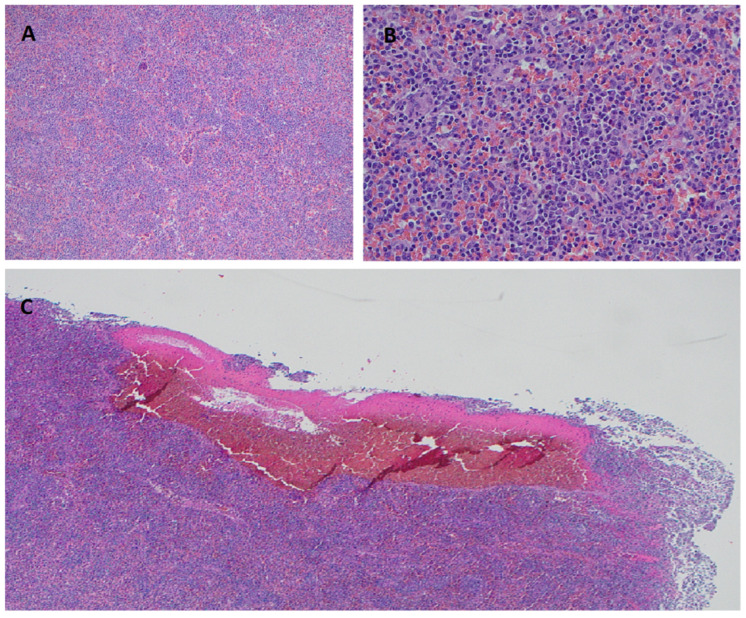
SDRPL Histomorphology on hematoxylin-eosin stained splenic tissue. Diffuse infiltration of splenic red pulp with small-to-medium sized lymphocytes and effacement of the white pulp at 100× (**A**) and 400× (**B**). Formation of pseudosinusoids/blood lakes caused by sinusoidal disruption at 40× (**C**).

The diagnosis of SDRPL is mainly based on the splenic histology and immunohistochemistry. However, the diagnosis can also be suggested by demonstrating BM (purely intra-sinusoidal pattern) and PB (predominance of villous lymphocytes) involvement with the appropriate immunophenotype, and the absence of other features which are more in keeping with entities in the differential diagnosis of SDRPL. However, a definite diagnosis may require a splenic biopsy or splenectomy. Gross examination of the spleen in SDRPL shows a diffuse enlargement without nodularity. Microscopic analysis (Figure 1) reveals a monomorphic infiltrate of small to medium sized lymphocytes with regular nuclei, clumped chromatin, and a pale cytoplasm diffusely involving the red pulp cords and sinusoids, with frequent effacement of the white pulp [8]. Focal plasmacytic differentiation can be present [9]. Small clusters of large cells and prominent nucleoli are infrequent. Sinusoidal disruption results in the collection of erythrocytes, surrounded by tumor cells, forming the characteristic blood lakes in some cases (Figure 1). True blood lakes tend to be smaller. and are lined by tumoral cells. In contrast, dilated sinusoids, which can also be present in some splenic lymphomas, are lined by littoral cells (CD8-positive/CD34-negative) and vascular endothelial cells (CD8-negative/CD34-positive). Expansion of the white pulp by follicular or nodular proliferation is absent, and this facilitates the correct categorization. BM can have interstitial, nodular, or intra-sinusoidal involvement [10], and the latter pattern is more specific. Lymphocytes often display broad-based, unevenly distributed, small cytoplasmic villi on BM aspirates and/or peripheral blood smears.

Immunohistochemistry (IHC) of the neoplastic cells shows positivity for CD19, CD20, and Bcl-2; they also show an usual negativity for CD5, CD10, CD23, CD43, Bcl-6, MUM1, cyclin D1, CD11c, CD25, CD123, and annexin A1. DBA.44 is positive in 20–90% of cases [7,8,10]. IgD and CD43 positivity has been described, although the former marker is more commonly associated with marginal zone B-cell lymphoma. A recent study showed cyclin D3 expression in 24 out of 33 SDRPL patients (72%) [11]. However, this marker is not readily available in most laboratories. Flow cytometry shows monoclonal B lymphocytes expressing surface immunoglobulin, which could be either biclonal (M and G or M and D) or monoclonal (G or M alone), and can detect CD103 expression in up to a third of cases [12]. IgVH somatic hypermutation analysis shows alterations in up to 70% of cases with the selective usage of V_H_3 and V_H_4 gene families [6,13]. Cytogenetic abnormalities, most commonly chromosome 7q deletion, trisomy 18, and partial trisomy 3q, can be detected in a third of SDRPL cases. About 10% of the cases have a complex karyotype, and TP53 alterations are rare, seen in 5–15% of cases [8,9].

A recent whole-exome sequencing (WES) study of 25 SDRPL patients identified CCND3 mutations in six cases (24%), whereas no mutations in NOTCH2 or BRAFV600E were reported [11]. Another recent study observed a distinct mutational landscape in 42 SDRPL samples when compared to 8 and 46 samples from HCL and SMZL patients, respectively [13]. WES of these three separate entities identified recurrent BCOR (BCL6 corepressor) mutations or losses in 10 of 42 SDRPL cases (24%), while it was rare in SMZL (1 of 46) and absent (zero of eight) in HCL. CCND3 mutations were also detected in 21% of SDRPL and 13% of SMZL cases. A BRAF V600E mutation was identified in all HCL samples (eight of eight) but only in 1 of 42 SDRPL and 1 of 46 SMZL samples. Mutations in the NOTCH (NOTCH2, NOTCH1 and SPEN) and NF-𝜅B pathways (KLF2, TNFAIP3 and MYD88) were also rare in SDRPL as opposed to SMZL samples. These findings can aid in the differential diagnosis of cases presenting with primary splenic lymphomas.

## 4. Differential Diagnosis

SDRPL shares certain clinicopathologic features with SMZL, HCL, and HCL-v, all of which can also present with splenomegaly and villous B lymphocytes in the PB (Table 1). Classical SMZL is differentiated from SDRPL and HCL-v by its nodular involvement of the white pulp with a frequent targetoid appearance, composed of a central zone with small lymphocytes surrounded by a peripheral zone of larger, paler marginal zone cells, which is more evident on Ki-67 immunostains. Aside from the previously mentioned IgD expression by immunohistochemistry, cytogenetic abnormalities are more common (up to 80%) than in SDRPL. Mainly deletion 7q (16–36%), trisomy 3q (10–36%), and trisomy 12q (6–25%) are described [14]. IgVH mutations are seen in about 50% of the cases, with predilection for V_H_1-2 gene usage, which is distinct from SDRPL. NOTCH2, which plays an important role in the development of marginal zone B-cells, is the most commonly mutated gene in SMZL (20–25%).

HCL and HCL-v are both characterized by a diffuse pattern of red pulp involvement, accompanied by atrophy of the white pulp, as seen in SDRPL. However, clinicopathologic features are very characteristic and usually allow segregation from SDRPL. HCL presents with significant pancytopenia in the majority of the cases and lymphocytosis is uncommon. HCL-v is often associated with lymphocytosis, typically at a higher level when compared to SDRPL. The immunophenotype of HCL is peculiar, and includes the expression of CD11c, CD25, CD103, CD123, TRAP, DBA.44, Annexin A1, and CD200, in addition to unspecific pan B-cell antigens, including CD19, CD20, and CD22. Moreover, almost all cases of HCL carry the BRAF V600E mutation. Furthermore, BM morphology may show focal or diffuse involvement, often with a characteristic neoplastic cytology demonstrating round/oval nuclei, and somewhat abundant well-demarcated cytoplasm (conferring a ‘fried-egg’ appearance), decorated by significant delicate reticulin fibrosis. This fibrotic background delineates each cell and results in the characteristic unyielding bone marrow aspirates (dry taps). Therefore, HCL rarely represents a diagnostic dilemma on the morphologic examination of BM biopsies.

Similarly, HCL-v, a distinct lymphoproliferative disorder that resembles HCL but is considered different by its cell of origin (activated late-stage B-cell vs. mature B-cell of unknown type in HCL), and usually shows features that allows for a categorical diagnosis. In contrast with HCL, the variant usually shows leukocytosis/lymphocytosis and a unique morphology, consisting of insignificant reticulin fibrosis, prominent nucleoli/prolymphocytoid features, blastic or convoluted nuclei, and/or absence of cytoplasmic circumferential villous projections. Furthermore, HCL-v displays an eponymous variant immunophenotype, which it shares with HCL’s positivity for CD11c and CD103, but is typically negative for CD25, CD123, TRAP, and annexin A1. In addition, it also lacks the BRAF V600E mutation, but may have activating mutations in MAP2K1, as reported in a fraction (48%) of cases [15].

## 5. Clinical Features

All patients present with stage IV disease involving BM and PB, and almost all patients have splenomegaly, which is frequently massive and can cause abdominal pain. B symptoms are not uncommon, and are reported in up to a third of patients [7]. Low level lymphocytosis is usually present. However, BM involvement and/or hypersplenism can lead to leukopenia and thrombocytopenia, which are usually mild. Anemia is rare, and absent in the vast majority of case series in the literature. SPEP can detect a monoclonal IgG or IgM spike in 5–10% of the patients. A few cases with cutaneous involvement, in the form of erythematous and pruritic plaques, have been reported [7]. Concurrent chronic hepatitis B infection has been described in two cases of SDRPL [16].

**Figure 2:**
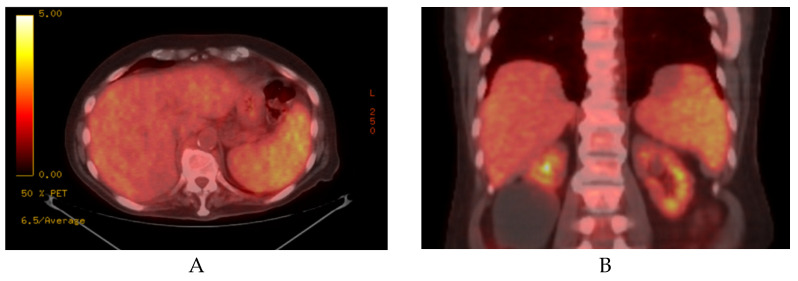
Axial (**A**) and coronal (**B**) sections of a PET/CT scan showing mild-moderate splenomegaly with increased FDG uptake in a patient with SDRPL.

Imaging studies, such as abdominal ultrasound and CT, often show a diffuse splenic enlargement without discrete splenic lesions. Mildly increased homogenous FDG-avidity in the spleen may be seen on PET/CT, as shown in Figure 2 [4]. Rarely, normal splenic size/appearance and FDG uptake is present [3].

Transformation to a large B-cell lymphoma with a high LDH level, Ki-67 index, and aggressive behavior has been described in rare cases [7,16,17], and transformation to B-cell prolymphocytic leukemia has also been published [18,19].

## 6. Treatment

Due to the rarity of this disease, there is a lack of retrospective or prospective clinical studies comparing different treatment options. Management strategies are usually extrapolated from regimens that are used for other types of primary splenic lymphomas, such as SMZL. Watchful waiting, splenectomy, and rituximab monotherapy are the most commonly utilized frontline treatment strategies for SDRPL. Treatment outcome data belong to individual case reports and case series. In a 17-patient case series, in which eleven patients (65%) underwent a frontline splenectomy and six patients underwent frontline chemotherapy, followed by splenectomy at 24, 30, 36, 80, and 168 months from the diagnosis, 5-year overall survival (OS) was reported as 93% [7]. Another study of 13 SDRPL patients who underwent splenectomy as a first line therapy showed a 2 year OS and a progression free survival (PFS) of 92% and 62%, respectively [9]. Although splenectomy can achieve durable remissions, it is not curative, since residual disease remains in the BM and PB compartments. In addition, splenectomy is associated with postoperative complications and infection risks, which are augmented in the elderly. Therefore, rituximab monotherapy is often recommended and well tolerated in older patients.

## 7. Clinical Vignette

A 72-year-old white male with multiple comorbidities presented with a 3 weeks history of extreme fatigue, a fever up to 102° F, drenching night sweats, early satiety and a weight loss of 10 lbs. CBC showed a WBC count of 15,000/mm^3^ with 60% lymphocytes, hemoglobin of 15 g/dL, and a platelet count of 102,000/mm^3^. Serum protein electrophoresis (SPEP) demonstrated an IgG kappa monoclonal gammopathy (2.4 g/dL). CT scan showed splenomegaly measuring 17.7 cm without any lymphadenopathy. PET scan revealed diffusely increased FDG uptake, which was limited to the spleen. A peripheral blood (PB) smear demonstrated atypical small mature lymphocytes, including villous forms. Since a lymphoid neoplasm was highly suspected, PB flow cytometry was ordered as the first diagnostic test. PB flow cytometry showed clonal lymphocytes that were positive for kappa light chain (bright), CD19, CD20, and CD22, and were negative for CD5, CD10, CD11c, CD23, CD138, and DBA.44. Bone marrow biopsy showed lymphoid aggregates suggestive of lymphomatous involvement. He underwent diagnostic and therapeutic splenectomy. The spleen showed a predominant red pulp expansion with tiny lymphoid nodules/aggregates, consisting of atypical small-to-medium sized B lymphocytes with minimally irregular nuclei and a varying degree of plasmacytic differentiation. Small blood lakes were identified. Immunohistochemistry revealed the neoplastic cells to be positive for CD20 and IgG, and negative for CD5, CD10, and Cyclin D1. A diagnosis of splenic diffuse red pulp small B-cell lymphoma (SDRPL) was made, based on the exclusion of MZL (which is typically positive for IgD and classically shows a white pulp predominant pattern), LPL (very unlikely in the absence of an IgM paraprotein) and HCL (unique histomorphology and immunophenotype). Patient’s symptoms resolved after splenectomy except for fatigue, which was later attributed to his cardiac comorbidities.

## 8. Summary

SDRPL is a rare type of indolent B-cell lymphoma involving the spleen, BM, and PB, which is more prevalent in older male patients (median age ~65 years-old). SDRPL often presents with massive splenomegaly, mild leukocytosis, and mild thrombocytopenia. The golden standard for diagnosis is a splenic tissue examination from splenectomy or biopsy. However, demonstration of typical histologic, immunophenotypic, and molecular findings in the BM and/or PB may also be diagnostic. Differentiating SDRPL from other types of primary splenic lymphomas, such as SMZL, HCL, and HCL-v may be challenging due to overlapping features. Ancillary studies, including PCR, cytogenetics, and next generation sequencing (NGS) may be necessary. Watch and wait is the most suitable strategy for asymptomatic patients due to the indolent nature of SDRPL. Symptomatic patients can achieve long remission with splenectomy or rituximab monotherapy. Chemotherapy should be reserved for disease progression.

## Figures and Tables

**Table 1:** Comparative clinicopathological and molecular features of SDRPL, SMZL, HCL, and HCL-v.

Character	SDRPL	SMZL	HCL	HCL-v
**Clinical features**				
Splenomegaly	Present	Present	Present	Present
B symptoms	∼1/3	∼1/4	Rare	Rare
**Laboratory findings**				
Pancytopenia	Rare	+	++	+
Lymphocytosis	+	+	Rare	++
**Histology**				
Spleen	Red pulp with blood lakes	White pulp	Red pulp with blood lakes	Red pulp with blood lakes
Bone marrow	Sinusoidal +/− interstitial, rarely nodular	Para-trabecular and sinusoidal	Interstitial to nodular/diffuse fibrosis	Interstitial and sinusoidal
Peripheral blood	Small, broad based villi	Small unevenly distributed villi	Abundant, long villi	Polar villi, prominent nucleoli
**Immunohistochemistry**				
CD20	++	++	++	++
CD5	−	−/+	−	−
CD10	−	−	−	−
CD23	−	−	−	−
Cyclin D3	++	−	−	−
Annexin I	−	−	++	−
DBA.44	+/−	+/−	++	++
IgG	+	+/−	+/−	+/−
CD11c	−	+	++	++
IgD	−	+/−	+/−	+/−
CD25	−	+	++	−
CD103	−	+/−	++	+
CD123	−	−	++	−
**Molecular studies**				
FISH	7q del, trisomy 18 and 3q	Trisomy 3, del 7q	Trisomy 5, del 5q	17p del
Genetic mutations	CCND3, BCOR	NOTCH2, KLF2	BRAF V600E	MAP2K1

+ indicates usually positive, − indicates usually negative, +/− indicates variably positive, ++ indicates mostly positive.

## References

[1] International Agency for Research on Cancer, World Health Organization (2008). WHO Classification of Tumours of Haematopoietic and Lymphoid Tissues.

[2] Swerdlow S.H., Campo E., Pileri S.A., Harris N.L., Stein H., Siebert R., Advani R., Ghielmini M., Salles G.A., Zelenetz A.D. (2016). The 2016 revision of the World Health Organization classification of lymphoid neoplasms. Blood.

[3] Saboo S.S., Krajewski K.M., O’Regan K.N., Giardino A., Brown J.R., Ramaiya N., Jagannathan J.P. (2012). Spleen in haematological malignancies: Spectrum of imaging findings. Br. J. Radiol..

[4] Shimizu-Kohno K., Kimura Y., Kiyasu J., Miyoshi H., Yoshida M., Ichikawa R., Niino D., Ohshima K. (2012). Malignant lymphoma of the spleen in Japan: A clinicopathological analysis of 115 cases. Pathol. Int..

[5] Li M., Zhang L., Wu N., Huang W., Lv N. (2013). Imaging Findings of Primary Splenic Lymphoma: A Review of 17 Cases in Which Diagnosis Was Made at Splenectomy. PLoS ONE.

[6] Traverse-Glehen A., Baseggio L., Callet-Bauchu E., Morel D., Gazzo S., Ffrench M., Verney A., Rolland D., Thieblemont C., Magaud J. (2008). Splenic red pulp lymphoma with numerous basophilic villous lymphocytes: A distinct clinicopathologic and molecular entity?. Blood.

[7] Kanellis G., Mollejo M., Montes-Moreno S., Rodriguez-Pinilla S.-M., Cigudosa J.C., Algara P., Montalban C., Matutes E., Wotherspoon A., Piris M.A. (2010). Splenic diffuse red pulp small B-cell lymphoma: Revision of a series of cases reveals characteristic clinico-pathological features. Haematologica.

[8] Medeiros L.J., Miranda R.N. (2018). Splenic Diffuse Red Pulp Small B-Cell Lymphoma. Diagnostic Pathology: Lymph Nodes and Extranodal Lymphomas.

[9] Martinez D., Navarro A., Martinez-Trillos A., Molina-Urra R., Gonzalez-Farre B., Salaverria I., Nadeu F., Enjuanes A., Clot G., Costa D. (2016). NOTCH1, TP53, and MAP2K1 Mutations in Splenic Diffuse Red Pulp Small B-cell Lymphoma Are Associated with Progressive Disease. Am. J. Surg. Pathol..

[10] Ponzoni M., Kanellis G., Pouliou E., Baliakas P., Scarfò L., Ferreri A.J., Doglioni C., Bikos V., Dagklis A., Anagnostopoulos A. (2012). Bone Marrow Histopathology in the Diagnostic Evaluation of Splenic Marginal-zone and Splenic Diffuse Red Pulp Small B-cell Lymphoma. Am. J. Surg. Pathol..

[11] Curiel-Olmo S., Mondéjar R., Almaraz C., Mollejo M., Cereceda L., Marès R., Derdak S., Campos-Martín Y., Batlle A., De Villambrosía S.G. (2017). Splenic diffuse red pulp small B-cell lymphoma displays increased expression of cyclin D3 and recurrent CCND3 mutations. Blood.

[12] Baseggio L., Traverse-Glehen A., Callet-Bauchu E., Morel D., Magaud J., Berger F., Salles G., Felman P. (2011). Relevance of a scoring system including CD11c expression in the identification of splenic diffuse red pulp small B-cell lymphoma (SRPL). Hematol. Oncol..

[13] Jallades L., Baseggio L., Sujobert P., Huet S., Chabane K., Callet-Bauchu E., Verney A., Hayette S., Desvignes J.-P., Salgado D. (2017). Exome sequencing identifies recurrent BCOR alterations and the absence of KLF2, TNFAIP3 and MYD88 mutations in splenic diffuse red pulp small B-cell lymphoma. Haematologica.

[14] Dufresne S.D., Felgar R.E., Sargent R.L., Surti U., Gollin S., McPhail E., Cook J.R., Swerdlow S.H. (2010). Defining the borders of splenic marginal zone lymphoma: A multiparameter study. Hum. Pathol..

[15] Waterfall J., Arons E., Walker R.L., Pineda M., Roth L., Killian J.K., Abaan O.D., Davis S., Kreitman R.J., Meltzer P.S. (2013). High prevalence of MAP2K1 mutations in variant and IGHV4-34–expressing hairy-cell leukemias. Nat. Genet..

[16] Kerbauy M.N., Fernandes C.M., Bezerra E.D., Lage L.A., Siqueira S.A.C., Pereira J. (2016). Splenic diffuse red-pulp small B-cell lymphoma associated with hepatitis B virus: A report of two cases. Sao Paulo Med. J..

[17] Wang R.-C., Medeiros L.J., Chang K.-C. (2018). Villous lymphocytes in splenic large B-cell lymphoma with diffuse red pulp infiltration. Int. J. Hematol..

[18] Hoehn D., Miranda R., Kanagal-Shamanna R., Lin P., Medeiros L.J. (2012). Splenic B-cell lymphomas with more than 55% prolymphocytes in blood: Evidence for prolymphocytoid transformation. Hum. Pathol..

[19] Cheng W.-Y., Zhu Y.-M., Cheng S., Chen Y.-S., Shen Y. (2017). Development of B-cell prolymphocytic leukemia in a patient with splenic diffuse red pulp small B-cell lymphoma. Leuk. Lymphoma.

